# Surface superconductivity in the type II Weyl semimetal TaIrTe_4_

**DOI:** 10.1093/nsr/nwz204

**Published:** 2019-12-16

**Authors:** Ying Xing, Zhibin Shao, Jun Ge, Jiawei Luo, Jinhua Wang, Zengwei Zhu, Jun Liu, Yong Wang, Zhiying Zhao, Jiaqiang Yan, David Mandrus, Binghai Yan, Xiong-Jun Liu, Minghu Pan, Jian Wang

**Affiliations:** 1 Department of Materials Science and Engineering, College of New Energy and Materials, China University of Petroleum, Beijing 102249, China; 2 International Center for Quantum Materials, School of Physics, Peking University, Beijing 100871, China; 3 School of Physics and Information Technology, Shaanxi Normal University, Xi’an 710119, China; 4 School of Physics, Huazhong University of Science and Technology, Wuhan 430074, China; 5 Wuhan National High Magnetic Field Center, Huazhong University of Science and Technology, Wuhan 430074, China; 6 Center of Electron Microscopy, State Key Laboratory of Silicon Materials, School of Materials Science and Engineering, Zhejiang University, Hangzhou 310027, China; 7 Department of Materials Science and Engineering, University of Tennessee, Knoxville, TN 37996, USA; 8 Department of Physics and Astronomy, University of Tennessee, Knoxville, TN 37996, USA; 9 Materials Science and Technology Division, Oak Ridge National Laboratory, Oak Ridge, TN 37831, USA; 10 Department of Condensed Matter Physics, Weizmann Institute of Science, Rehovot 7610001, Israel; 11 CAS Center for Excellence in Topological Quantum Computation, University of Chinese Academy of Sciences, Beijing 100190, China; 12 Beijing Academy of Quantum Information Sciences, Beijing 100193, China; 13 Collaborative Innovation Center of Quantum Matter, Beijing 100871, China

**Keywords:** surface superconductivity, Weyl semimetal, topological superconductivity

## Abstract

The search for unconventional superconductivity in Weyl semimetal materials is currently an exciting pursuit, since such superconducting phases could potentially be topologically non-trivial and host exotic Majorana modes. The layered material TaIrTe_4_ is a newly predicted time-reversal invariant type II Weyl semimetal with the minimum number of Weyl points. Here, we report the discovery of surface superconductivity in Weyl semimetal TaIrTe_4_. Our scanning tunneling microscopy/spectroscopy (STM/STS) visualizes Fermi arc surface states of TaIrTe_4_ that are consistent with the previous angle-resolved photoemission spectroscopy results. By a systematic study based on STS at ultralow temperature, we observe uniform superconducting gaps on the sample surface. The superconductivity is further confirmed by electrical transport measurements at ultralow temperature, with an onset transition temperature (*T*_c_) up to 1.54 K being observed. The normalized upper critical field h*(*T/T*_c_) behavior and the stability of the superconductivity against the ferromagnet indicate that the discovered superconductivity is unconventional with the *p*-wave pairing. The systematic STS, and thickness- and angular-dependent transport measurements reveal that the detected superconductivity is quasi-1D and occurs in the surface states. The discovery of the surface superconductivity in TaIrTe_4_ provides a new novel platform to explore topological superconductivity and Majorana modes.

## Teaser

Surface superconductivity detected in Weyl semimetals provides a new platform to explore topological superconductivity and Majorana modes.

## INTRODUCTION

Weyl semimetals, which possess nodal points in the bulk and Fermi arc states on the surface, have generated considerable research interest in recent years [1–12]. The chirality of Weyl fermions is responsible for a few novel transport phenomena, such as the chiral anomaly. On the surface of Weyl semimetals, universal signatures of topological Fermi arcs in quasi-particle interference were theoretically predicted [13] and experimentally observed by scanning tunneling microscopy (STM) [14–16]. Moreover, the theoretical studies have shown that the presence of superconductivity in Weyl semimetals may lead to many novel topological phases, including the time-reversal invariant topological superconductor [17], Fulde–Ferrell–Larkin–Ovchinnikov superconductors [18–20] and chiral non-Abelian Majorana fermions protected by second Chern numbers [21]. These predictions suggest that turning a Weyl semimetal into a superconducting state may provide a promising way to explore topological superconductivity and Majorana modes, which can be applied to topological quantum computation [22–24].

Experimentally, superconductivity has been observed in both type I and type II Weyl semimetals, such as tip-induced superconductivity on TaAs [25], pressure-induced superconductivity on TaP [26], as well as T_d_ phase WTe_2_ (pressure driven) [27,28] and MoTe_2_ crystals (without pressure) [16,29]. However, in these Weyl semimetals, the number of Weyl points is 8 or even 24, more than the minimal number of Weyl points allowed for a time-reversal invariant Weyl semimetal, which leads to complicated band structures and hinders further studies. Therefore, to observe superconductivity in simpler Weyl semimetals possessing the minimal number of Weyl points is highly desired.

Following the first-principle calculations by K. Koepernik *et al*. [30], TaIrTe_4_ hosts only four well-separated Weyl points, which is the minimum number in a Weyl semimetal with time-reversal symmetry. The Fermi arcs connecting Weyl nodes of opposite chirality in TaIrTe_4_ extend to about ^1^/_3_ of the surface Brillouin zone in the *b* direction. This large momentum-space separation makes TaIrTe_4_ quite favorable for exploring the Fermi arcs spectroscopically and the important transport properties. The exotic surface states supporting the quasi-1D Fermi arcs have been observed by angle-resolved photoemission spectroscopy (ARPES) [31]. Fermi arcs as well as Weyl nodes in the bulk of TaIrTe_4_ have been identified directly by pump-probe ARPES [32]. The Weyl points and Fermi arcs are found to live at 50–100 meV above Fermi energy. If the non-centrosymmetric Weyl material TaIrTe_4_ can be superconducting, it would stimulate further investigations on the superconductivity in topological materials and long-sought-after topological superconductors.

In this work, we perform scanning tunneling microscopy (STM) and spectroscopy (STS), and electrical transport studies of the ternary compound TaIrTe_4_ single crystal down to 0.06 K with a high magnetic field up to 54.5 T. The Fermi arc surface states and superconducting gap are discovered by STM and STS studies at ultralow temperatures. The detected unconventional superconductivity is further verified to only occur on the surface of TaIrTe_4_ by electrical transport measurements. The observed unconventional surface superconductivity is found to exhibit quasi-1D and topologically non-trivial characteristics, which demonstrate that TaIrTe_4_ is a unique candidate of topological superconductor.

## RESULTS

### Sample characterization of TaIrTe_4_ crystals

**Figure 1.**
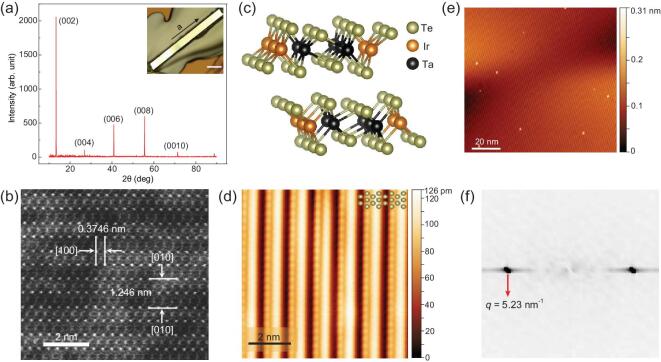
Characterization of Weyl semimetal TaIrTe_4_. (a) The XRD pattern from the basal surface of TaIrTe_4_ only shows (002)_n_ reflections, which indicates the measured crystal plane of the crystal is the (001) plane. Inset: optical image of a typical TaIrTe_4_ single crystal. The scale bar is 200 μm. (b) HRSTEM image of the TaIrTe_4_, showing atomic structure. (c) Schematics of crystal structure of TaIrTe_4_. (d) and (e) STM images of the fresh cleaved surface of TaIrTe_4_ with the setting parameters of *V*_bias_ = 25 mV, *I*_set_ = 300 pA and *V*_bias_ = 2.5 V, *I*_set_ = 20 pA, respectively. (f) Fast Fourier transform image of (d).

Single crystals of TaIrTe_4_ were synthesized from excess Te flux. The crystal has a needle-like morphology and grows preferentially along the [100] direction (the length direction). The width direction is along the [010] and the cleavage surface of the crystal is the (001) plane. The high crystalline quality of the sample was confirmed by X-ray diffraction (XRD), high-resolution scanning transmission electron microscopy (HRSTEM) and STM. Figure 1a shows the XRD from a TaIrTe_4_ crystal oriented with the scattering vector perpendicular to the (001) plane. The inset is the morphology of a representative crystal looking down from the [001] direction. The atomic HRSTEM image (Fig. 1b) and selected area electron diffraction pattern (Fig. S1 in the online supplementary material) further confirm the crystalline property of our TaIrTe_4_ crystals. The obtained lattice parameters are ***a*** = 0.375 nm, ***b*** = 1.246 nm, ***c*** = 1.304 nm, which agree with the previous report on TaIrTe_4_ [33]. STM investigation shows uniform large-scaled periodical 1D stripes on a cleaved TaIrTe_4_ surface. Several bright spots appear on the flat Te terrace, which can be attributed to some adatoms from the upper Te plane left on the terrace during cleaving. From a zoom-in image (Fig. 1d), a unidirectional stripe was observed. The fast Fourier transform (FFT) in Fig. 1f used to calibrate the modulation of the 1D pattern, reveals the periodicity of stripe is about 1.2 nm in real space. This is in good agreement with lattice parameter of the ***b*** direction (12.421 Å), which suggests no reconstruction in the ***b*** direction.

### Fermi arc surface states and superconducting gap detected by STM/STS

Quasiparticle interference (QPI), based on spectroscopic-imaging STM, has shown success in identifying the topological surface states of topological insulator [34,35] and topological Fermi arc states of Weyl semimetals TaAs [36], MoTe_2_ [14], MoTe_2-x_S_x_ [16] and Mo_0.66_W_0.34_Te_2_ [37]. In the surface Brillouin zone, the extremal pairs of ***k****_i_* and ***k****_f_* on a 2D constant-energy contour, where ***k****_i_* and ***k****_f_* are the initial and final wavevectors, contribute dominantly to the spatial interference pattern of the local density of states. The features in Fourier transform of d*I*/d*V* mapping correspond to the scattering vector ***q*** =** *k****_i_* − ***k****_f_* of the extremal pairs.

**Figure 2.**
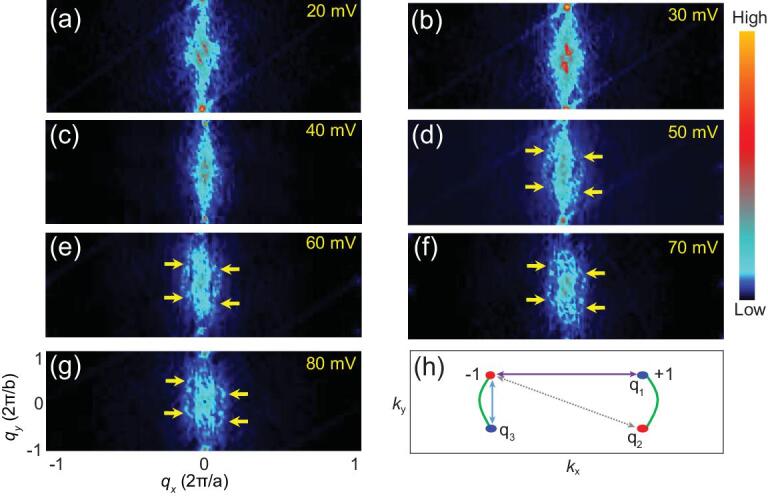
Fermi arc states of TaIrTe_4_ detected by STS. (a–g) Fourier transform of d*I*/d*V* maps at indicated energies. All maps were taken with set point of 250 pA. The resolution is 512 × 512 pixels. Yellow arrows indicate the interference pattern due to topological surface states. (h) Scattering geometry of Fermi arcs in *k* space.

To detect Fermi arc states in our TaIrTe_4_ single crystals, STS mappings were performed at the (001) surface of TaIrTe_4_ single crystals. Figure S2b–h in the online supplementary material shows d*I*/d*V* mappings taken with various biases from 20 meV to 80 meV. Figure 2a–g shows the FFT of the d*I*/d*V* maps between 20 meV and 80 meV. Four arcs located inside the first Brillouin zone were revealed by the QPI imaging at an energy of 80 meV (Fig. 2g). For a pair of topological Fermi arcs, three scattering wavevectors (Fig. 2h), labelled ***q***_1_, ***q***_2_ and ***q***_3_, might be expected to appear in QPI. Among them, ***q***_2_ is forbidden due to the requirement of the time-reversal symmetry in the system [30]. ***q***_3_ does not correspond to the observed features discussed above because this vector has very small length in *k* space and stays very close to the center. The scattering wavevectors should generate visible features of four arcs. Such features are clearly resolved at 80 mV in our experiments as indicated by yellow arrows in Fig. 2g, and become obscure at lower energies (50–70 meV), eventually vanishing below 40 meV. Previously theoretical study has reported that the Fermi arc locates at a narrow energy range between 50 and 82.7 meV in TaIrTe_4_ [30]. As the energy location moves upward from 50 meV, the Fermi arc partially separates out from bulk bands and completely appears at the energy of Weyl nodes (82.7 meV). The mixing of Fermi arc and bulk band at lower energies will lead to the obscureness of Fermi arc imaging, which is consistent with our experimental observation. This is direct and strong experimental evidence for the existence of the topological surface states.

**Figure 3.**
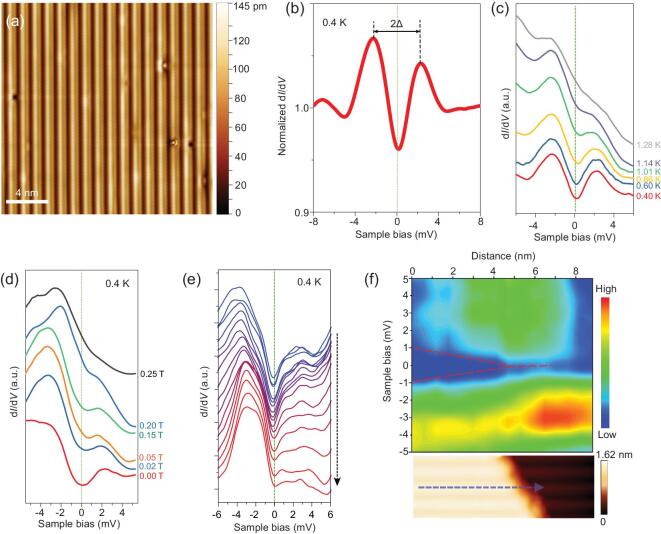
The superconductivity in TaIrTe_4_ detected by STM/STS_._ (a) The typical STM topographic image of the cleaved surface of TaIrTe_4_ (temperature: 4 K; bias voltage: −50 mV; tunneling current: 300 pA; 20 × 20 nm^2^), showing quasi-1D structure. (b) The normalized differential conductance d*I*/d*V* spectrum measured on the terrace of TaIrTe_4_ surface at 0.4 K, showing a superconducting gap, with the value of 2.1 meV. (c) Temperature dependence of d*I*/d*V* spectra from 0.4 K to 1.28 K. Spectra measured at different temperatures are shifted vertically for clarity. (d) Magnetic field dependence of d*I*/d*V* spectra from 0 T to 0.25 T at 0.4 K. (e) From top to bottom: the d*I*/d*V* spectra acquired along 1D atomic row at 0.4 K by crossing a terrace edge shown in lower panel of (f). (f) A color plot of the spectroscopic survey measured along the blue dashed line shown in lower panel. Lower: the STM image shows the 1D atomic row with a broken end. All d*I*/d*V* tunneling spectra are measured with a bias voltage of −10 mV and a tunneling current of 500 pA. The bias modulation is set at 150 μV.

**Figure 4.**
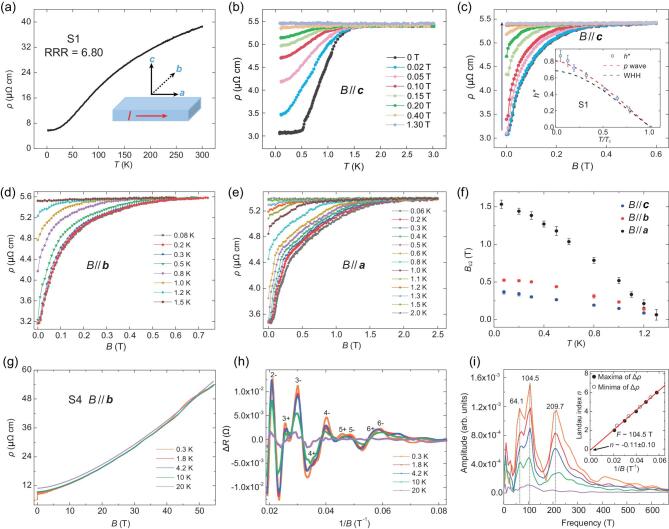
Electric transport properties of TaIrTe_4_ single crystal (Sample 1, S1) showing quasi-1D superconductivity. (a) Resistivity as a function of temperature between 2 K and 300 K. Inset shows schematic structure of crystal orientation in TaIrTe_4_ samples. (b) *ρ*(*T*) curves at different perpendicular magnetic fields (*B*//***c*** axis) from 0 T to 1.30 T. At 0 T, the sample resistivity begins to drop at 1.54 K (*T*_c_). (c) *ρ*(*B//****c***) curves at various temperatures at 0.08 K, 0.2 K, 0.3 K, 0.5 K, 0.8 K, 1.0 K, 1.2 K, 1.5 K and 2.0 K. The arrow indicates the increasing temperature. Inset: normalized upper critical field h* = *B*_c2_/[*T*_c_(−d*B*_c2_/d*T*|*_T_*=*_T_*_c_)] as a function of normalized temperature *T*/*T*_c_. The red dashed line indicates the expectation for a polar *p*-wave state. The black dashed line indicates the WHH theory for *s*-wave superconductor. (d) *ρ*(*B*//***b***) curves and (e) *ρ*(*B//****a***) curves at various temperatures. (f) Onset critical magnetic fields *B*_c2_ for *B*//***b***, *B*//***a*** and *B*//***c*** as a function of temperature *T*. (g) Quantum oscillations in TaIrTe_4_ single crystals (Sample 4, S4) at *B*_[010]_ direction. Magnetic field (up to 54.5 T)-dependence of resistivity at different temperatures with magnetic field perpendicular to the *ac* plane (*B//****b***). (h) The oscillatory component of Δ*ρ* extracted from *ρ* by subtracting a polynomial background, as a function of 1/*B* at various temperatures. (i) FFT analysis with two major frequencies (64.1 T and 104.5 T) for Δ*ρ* vs. 1/*B* in (h). Inset: Landau index (*n*) as a function of 1/*B*. *B*_lim_ is estimated to be 95.32 T.

Figure 3a gives the typical STM topographic image of the cleaved surface obtained at the bias of −20 mV and at a temperature of 4 K. Periodical atomic chains along *a* direction are clearly observed on the surface, confirming the quasi-1D characteristic of TaIrTe_4_. Compared with 4 K, the crystal structure at 0.4 K remains undistorted, which excludes the possibility of structure phase transition occurring at low temperatures. The d*I*/d*V* spectrum taken at 0.4 K displays a clear signature of superconducting gap with two conductance peaks at gap edges, as shown in Fig. 3b. The spectrum exhibits a superconducting gap (Δ) of 2.1 meV defined by half the distance between the two conductance peaks. The superconducting gap is uniform on the whole cleaved surface (see Figs S3 and S4 in the online supplementary material). After macroscopically changing the locations of STM scanning, similar topographic images and superconductivity were observed reproducibly. Figure 3c shows the temperature evolution of d*I*/d*V* spectra measured from 0.4 K to 1.28 K. As the temperature increases, the dip at zero bias is reduced and the gap almost vanishes near 1.28 K. The ratio Δ(0)/*k*_B_*T*_c_ (*k*_B_ is the Boltzmann constant) is estimated to be ∼19.05, which is much larger than that of weak coupling Bardeen–Cooper–Schrieffer (BCS) superconductors and reminiscent of the possibility of topological superconductivity [16,38,39]. The magnetic field dependences of d*I*/d*V* spectra are shown in Fig. 3d. The superconducting gap decreases with the increasing field and almost vanishes near 0.25 T, exhibiting the typical feature of superconductivity. Both critical values (1.28 K and 0.25 T) match well with the results of our transport measurements (Fig. 4). Furthermore, we locate a terrace edge that is perpendicular with the direction of 1D atomic rows and perform a line spectroscopic survey along a 1D atomic row by crossing the broken end (blue dashed line in lower panel of Fig. 3f). The d*I*/d*V* spectra along the 1D Ta-Ir chain show that the superconducting gap becomes smaller and shallower by approaching the broken end and finally almost vanishes (Fig. 3e). It is worth mentioning that superconductivity can be observed on every terrace of the sample. The crucial dependence of the superconductivity on defects in the Ta-Ir chains again suggests that the pairing order might be unconventional, in contrast to the conventional *s*-wave order that is stable against defects.

### Electrical transport evidence of quasi-1D superconductivity and quantum oscillations

To further demonstrate and explore the observed superconductivity in TaIrTe_4_ single crystals, transport measurements at ultralow temperature were performed at the (001) surface of TaIrTe_4_ single crystals. The TaIrTe_4_ single crystal samples were cleaved to a smooth and fresh surface for transport measurements. More than 10 samples are studied and all samples exhibit consistent results. Figure 4a shows the resistivity of Sample 1 (S1) as a function of temperature (*T*) from 2 K to 300 K. The resistivity exhibits metallic-like behavior and tends to saturate at 10 K with a residual resistivity ratio (RRR) of 6.8 (the resistivity at room temperature over the resistivity at 2 K). Interestingly, upon further cooling, an evident resistivity drop appears at about 1.54 K

(Fig. 4b). When applying a perpendicular magnetic field (*B*//***c*** axis), the resistivity drop shifts to lower temperatures as the field increases and is completely suppressed at around 0.4 T. This is a typical superconducting behavior although no zero resistance is observed down to 0.06 K and the proportion of resistivity drop is ∼44% (Fig. 4b). Magnetotransport measurements for the *B*//***c*** axis (Fig. 4c), *B*//***b*** axis (Fig. 4d) and *B*//***a*** axis (Fig. 4e) were carried out at various temperatures from 0.08 to 2.0 K. It is evident that superconductivity at the *B*//***a*** axis varies differently from the other two directions. For example, *B*_c2_ is around 0.5 T at 0.1 K for both *B*//***c*** axis and *B*//***b*** axis, substantially smaller than *B*_c_ > 1.5 T for the *B*//***a*** axis situation (Fig. 4f). This agrees well with the observed *B*_c2_ (0.25 T at 0.4 K for the *B*//***c*** axis) from STS measurements (see Fig. 3d). Since zigzag Ta-Ir chains are along the ***a*** direction of TaIrTe_4_, the difference of *B*_c2_ may originate primarily from the anisotropy of the sample, which causes quasi-1D superconductivity [40,41]. Besides the observed anisotropic superconductivity, when the temperature is above *T*_c_, the pronounced anisotropic magnetoresistance (MR) at 2 K up to 15 T is also detected, which further confirms the anisotropic characteristic of TaIrTe_4_, as shown in Fig. S5 in the online supplementary material. For quasi-1D superconductors, below *T*_c_ phase slips can give rise to broad superconducting transition with the residual resistance [42], which may explain our observations. Another possible scenario is that the superconductivity occurs in the surface states that are helical states for a time-reversal invariant Weyl semimetal with dispersions along the ***a***-axis, leading to the quasi-1D *p*-wave superconducting phases. Similar resistivity drops are observed in two other TaIrTe_4_ samples, with onset *T*_c_ (where resistivity starts to drop) from 1.19 K to 1.38 K (Fig. S6 in the online supplementary material, consistent with the *T*_c_ ∼1.28 K obtained from STS results), confirming the observed superconductivity in TaIrTe_4_ crystals. Further measurements show that the *T*_c_ of different regions in the same TaIrTe_4_ sample exhibits consistent superconducting behavior, which excludes the macroscopic superconducting phase separation in TaIrTe_4_ crystals (Fig. S7 in the online supplementary material). The reduced critical field h* equals *B*_c2_/[*T*_c_(−d*B*_c2_/d*T*|*_T_*=*_T_*_c_)], which is calculated to compare with known models for *s*-wave superconductors (Werthamer–Helfand–Hohenberg theory, WHH, h*(0) ≈ 0.7 [43]) and spin-triplet *p*-wave superconductors (h*(0) ≈ 0.8 [44]). *B*_c2_ is defined as the field above which the TaIrTe_4_ sample becomes the normal state. Obviously, the h*(*T*/*T*_c_) relation is close to that of a polar *p*-wave state, suggesting the possibility of unconventional superconducting pairing symmetry in TaIrTe_4_ as shown in the inset of Fig. 4c. Critical current (*I*_c_) is another key feature of superconductors. Figure S8a and b in the online supplementary material depicts *R*(*I*) characteristics of Sample 3 (30 μm thick) at different temperatures and magnetic fields. At 0.3 K and 0 T, as the current increases, the sample is gradually tuned from superconducting state to normal state. The *I*_c_ is suppressed by both temperature and magnetic field, which provides further evidence of superconductivity in TaIrTe_4_. In addition, the existence of *p*-wave superconductivity is backed by the essential symmetry consideration, in which both the bulk and surface of the studied material break the inversion symmetry and thus allow the spin-triplet pairing [45].

We also investigate the MR at ultrahigh pulsed magnetic field. Figure 4g presents the magneto-resistivity of Sample 4 (S4) up to 54.5 T at various temperatures from 0.3 K to 20 K. At 0.3 K, superconducting drop below 0.5 T coexisting with pronounced Shubnikov-de Haas (SdH) oscillations is observed. By subtracting a polynomial background, the oscillatory components Δ*ρ* vs. 1/*B* at different temperatures are plotted in Fig. 4h, with the Δ*ρ* oscillations periodic in 1/*B*. In Fig. 4i, the oscillatory components Δ*ρ* are analyzed by employing FFT at various temperatures from 0.3 K to 20 K. The FFT spectra exhibit two oscillating frequencies at 64.1 T and 104.5 T. The second harmonics 209.7 T, ∼2 times 104.4 T, is likely due to spin splitting. We use two independent parameters to fit the effective mass m*. The m*∼0.349*m*_e_ and m*∼0.412*m*_e_ are obtained for 64.1 T and 104.5 T from the temperature dependence of the oscillation amplitudes fitted by the Lifshitz Kosevich formula. The inset of Fig. 4i is the plot of the Landau index *n* vs. 1/*B*, from which the quantum limit field is estimated to be 95.32 T. The maxima of the Δ*ρ* are assigned to be the integer indices (solid circles) and the minima of Δ*ρ* are plotted by open circles in the diagram as half-integer indices. A linear extrapolation of the *n* vs. 1/*B* plot gives the intercept value close to −0.11. The observed results are further confirmed in Sample 5 (S5) (Fig. S9 in the online supplementary material). The evolution of the Fermi surface in different directions can be revealed by the angular-dependent magnetic quantum oscillations (see Fig. S10 in the online supplementary material). The angle-resolved SdH FFT peaks from the *B*_[010]_ to *B*_[001]_ axis give the complexity and anisotropy of the Fermi surface, similar to the previous report by torque measurements [46].

### Evidence for surface superconductivity

**Figure 5.**
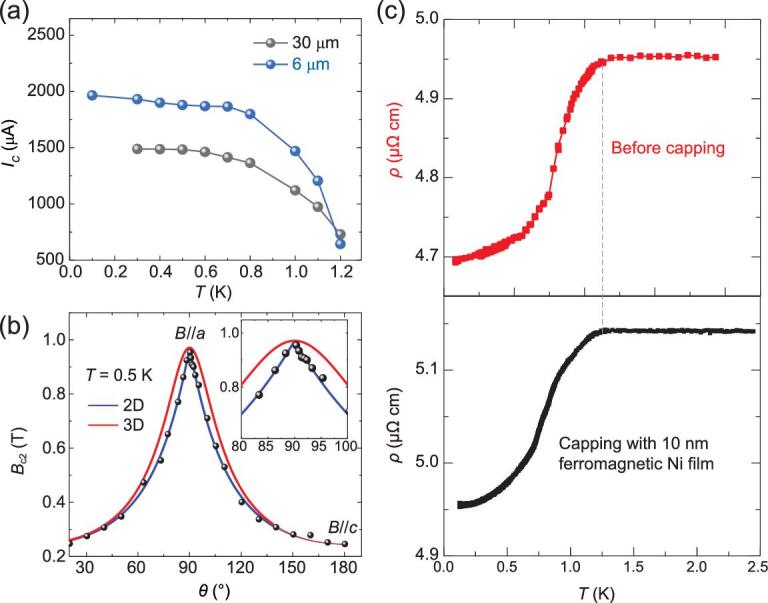
Evidences for unconventional surface superconductivity on TaIrTe_4_. (a) Temperature dependence of *I*_c_ of two TaIrTe_4_ samples with two different thicknesses (30 μm, 6 μm). These samples were obtained by mechanical exfoliation from one sample (Sample 3). (b) Angular dependence of the upper critical field *B*_c2_ at 0.5 K. The blue and red lines are the theoretical representations of *B*_c2_(θ) using the 2D Tinkham formula ${( {{{\rm{H}}_{{\rm{C2}}}}( {\rm{\theta }} ){\rm{sin\theta /H}}_{{\rm{C2}}}^{{\rm{//}}}} )^{\rm{2}}}{\rm{ + \ }}| {{{\rm{H}}_{{\rm{C2}}}}( {\rm{\theta }} ){\rm{cos\theta /H}}_{{\rm{C2}}}^ \bot } |{\rm{ = \ 1\ }}$and the 3D anisotropic mass model ${{\rm{H}}_{{\rm{C2}}}}\ ( {\rm{\theta }} ){\rm{ = H}}_{{\rm{C2}}}^{{\rm{//}}}{\rm{/}}{( {{\rm{si}}{{\rm{n}}^{\rm{2}}}{\rm{\theta + }}{{\rm{\gamma }}^{\rm{2}}}{\rm{co}}{{\rm{s}}^{\rm{2}}}{\rm{\theta }}} )^{{\rm{1/2}}}}$ with ${\rm{\gamma \ = H}}_{{\rm{C2}}}^{{\rm{//}}}\ {\rm{/H}}_{{\rm{C2}}}^ \bot$, respectively. The inset shows a close-up of the region around 90°. (c) Superconductivity of TaIrTe_4_ single crystal before and after capping with 10 nm-thick ferromagnetic Ni film on surface. The dashed gray line marks the temperature of onset *T*_c_.

The low proportion of resistivity drop in TaIrTe_4_ indicates the tiny superconducting volume fraction, and does not support the bulk superconductivity. Our STS results show a uniform superconducting gap on the surface of TaIrTe_4_. More importantly, it is found that the *I*_c_ remains the same magnitude when reducing the thickness of Sample 3 from 30 μm to 6 μm (see Fig. 5a and supplementary Fig. S8). In thickness control experiments, both the sample width (120 μm) and length (the distance between two voltage electrodes: 560 μm for 6 μm-thick sample and 590 μm for 30 μm-thick sample) are nearly consistent. This observation provides strong evidence of the surface superconductivity, rather than the bulk superconductivity, since the *I*_c_ of a bulk superconductor decreases proportionately as the thickness decreases. Note that the surface of TaIrTe_4_ is sensitive to atmosphere environment and the degree of surface oxidation is not exactly the same for different samples when carrying out transport measurements. Thus, the 25% discrepancy in *I*_c_ should be from the different degradation degrees of sample surface as the surface of the 6 μm-thick sample is fresher than the 30 μm-thick sample. Figure 5b shows the angular dependence of the upper critical field *B*_c2_ (*θ* represents the angle between the ***c*** axis and applied magnetic field directions in the ***ac*** plane) at 0.5 K. A cusp-like peak is clearly resolved at *θ* =* 9*0° (***B***//***a***) and is qualitatively distinct from the 3D mass model but can be described by the 2D Tinkham model. Such behavior further suggests that the superconductivity in TaIrTe_4_ is from the surface. These results fully exclude the scenario of the bulk superconductivity and support the surface superconductivity explanation. We notice that the $\gamma = {{H_{C2}^{//}}/{H_{C2}^ \bot}}$ value is smaller compared with typical 2D superconductors [47,48], which could be attributed to the quasi-1D modulation on the surface superconductivity [41].

The suppression of the superconductivity close to the terrace edge as shown in Fig. 3f is consistent with the surface superconductivity of the Fermi arc states. The terrace edge serves as the strong dislocation on the sample surface. Nearby the terrace edge the Fermi arc surface states are pushed into the deeper layers from the outermost layer, leading to a small or vanishing local density of states on the edge. For this reason, the superconductivity can be naturally suppressed. Due to the helical behavior of the Fermi arc states, the surface superconductivity is potentially *p*-wave type and topologically non-trivial. This *p*-wave feature can be further confirmed by depositing a 10 nm-thick ferromagnetic Ni film on the surface of bulk TaIrTe_4_. The MR hysteresis of TaIrTe_4_ with Ni film indicates the magnetic property of the deposited Ni film (Fig. S11 in the online supplementary material). Interestingly, the magnetic Ni film has little effect on the onset *T*_c_ of TaIrTe_4_ (Fig. 5c), which supports the *p*-wave-like or topological superconductivity from the surface state together with the fitting for critical field vs. temperature behavior, suppression of the superconductivity at the terrace edge, and the detected Fermi arc surface state.

## CONCLUSION

In conclusion, we have observed the novel superconductivity in type II Weyl semimetal TaIrTe_4_ by both low temperature STM/STS and transport studies. The uniform superconducting gap on the sample surface, residual resistance below *T*_c_, nearly thickness-independent ultralow critical current, and anisotropic upper critical field behavior indicate that the superconductivity occurs in the surface states. Moreover, the edge-sensitive superconducting gap, the critical field vs. temperature behavior, the topological Fermi arc surface states, and the stability of the superconductivity against the magnetization support the *p*-wave-like topological nature of the quasi-1D superconductivity. Our results suggest that TaIrTe_4_ is a promising new topological superconductor candidate.

## METHODS

### STM and STS measurement

Samples were cleaved *in situ* at room temperature under a vacuum with pressure better than 1 × 10^−10^ torr. The cleaved sample was quickly transferred into a Unisoko-1300 STM system for ultralow temperature measurements down to 0.4 K.

### Transport measurement

The transport measurements were carried out in a PPMS-16 system (Quantum Design), a pulsed high magnetic field system at Wuhan National High Magnetic Field Center and anisotropic upper critical field at a dilution refrigerator with vector magnet (Leiden CF450). For electrical transport measurements of the TaIrTe_4_ samples on a (001) plane, the standard four-probe or Hall structure configuration is used. The electric current is always applied parallel to the (001) plane along the ***a*** axis in our studies. Two silver paste current electrodes (I+ and I−) are pressed on both ends and across the entire width of the sample, so that the current can homogeneously go through the sample in the length direction [100]. The other two silver paste electrodes are pressed in the middle of the crystal as voltage probes. For magnetoresistance (or Hall resistivity) measurements, any additional Hall (or resistive) voltage signals due to the misalignment of the voltage leads have been corrected by reversing the direction of the magnetic field.

## Supplementary Material

nwz204_Supplemental_File
