# Supplementary material for: Surface superconductivity in the type II Weyl semimetal TaIrTe4
Source: Natl Sci Rev. 2019 Dec 16;7(3):579–87. doi: 10.1093/nsr/nwz204 (PMC8288950; doi:10.1093/nsr/nwz204)
Supplement: nwz204_Supplemental_File [file nwz204_supplemental_file.pdf]

SUPPLEMENTARY INFORMATION

Surface superconductivity in the type II Weyl semimetal $TaIrTe_4$

Ying Xing[1,2†], Zhibin Shao[3,†], Jun Ge[2], Jiawei Luo[2], Jinhua Wang[4,5], Zengwei Zhu[4,5], Jun Liu[6], Yong Wang[6], Zhiying Zhao[7,11], Jiaqiang Yan[7,8], David Mandrus[7,8], Binghai Yan[9], Xiong-Jun Liu[2,10,12,13,*], Minghu Pan[3,4,*], Jian Wang[2,10,12,13,*]

[1] *Department of Materials Science and Engineering, School of New Energy and Materials, China University of Petroleum, Beijing 102249, China;*

[2] *International Center for Quantum Materials, School of Physics, Peking University, Beijing 100871, China;*

[3] *School of Physics and Information Technology, Shaanxi Normal University, Xi'an 710119, China.*

[4] *School of Physics, Huazhong University of Science and Technology, Wuhan 430074, China;*

[5] *Wuhan National High Magnetic Field Center, Huazhong University of Science and Technology, Wuhan 430074, China;*

[6] *Center of Electron Microscopy, State Key Laboratory of Silicon Materials, School of Materials Science and Engineering, Zhejiang University, Hangzhou, 310027, China;*

[7] *Department of Materials Science and Engineering, University of Tennessee, Knoxville, Tennessee 37996, USA;*

[8] *Materials Science and Technology Division, Oak Ridge National Laboratory, Oak Ridge, Tennessee 37831, USA;*

[9] *Department of Condensed Matter Physics, Weizmann Institute of Science, Rehovot, 7610001, Israel;*

[10] *CAS Center for Excellence in Topological Quantum Computation, University of Chinese Academy of Sciences, Beijing 100190, China;*

[11] *Department of Physics and Astronomy, University of Tennessee, Knoxville, TN 37996, USA;*

[12] *Beijing Academy of Quantum Information Sciences, West Bld. #3, No. 10 Xibeiwang East Rd., Haidian District, Beijing 100193, China;*

[13] *Collaborative Innovation Center of Quantum Matter, Beijing 100871, China*

†These authors contributed equally to this work.

*e-mail: jianwangphysics@pku.edu.cn (J.W.); minghupan@hust.edu.cn (M.P.); xiongjunliu@pku.edu.cn(X.L.)

Figures

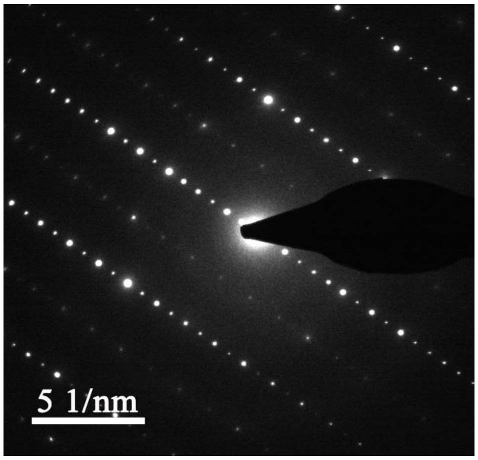

FIG. S1. Electron diffraction image looking down the [100] zone axis showing the reciprocal lattice of $TaIrTe_4$.

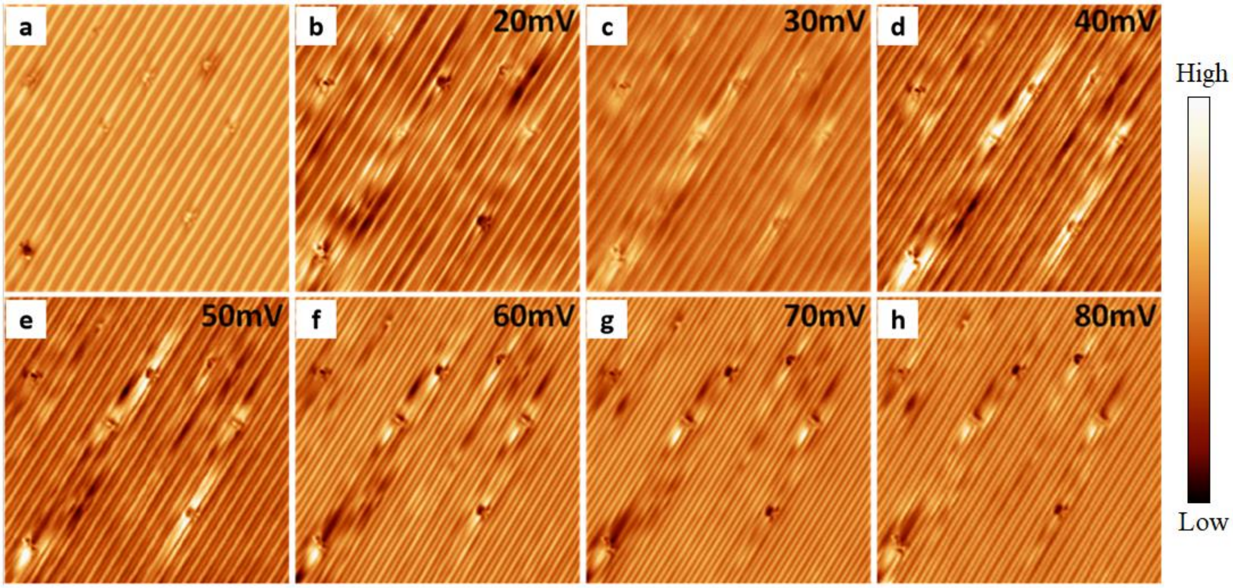

FIG. S2. (a) Topography of a chosen surface 40 nm $\times$ 40 nm and (b)- (h) the corresponding differential conductance mappings at different biases (I_{set}= 250 pA, bias modulation amplitude of 2.4 mV).

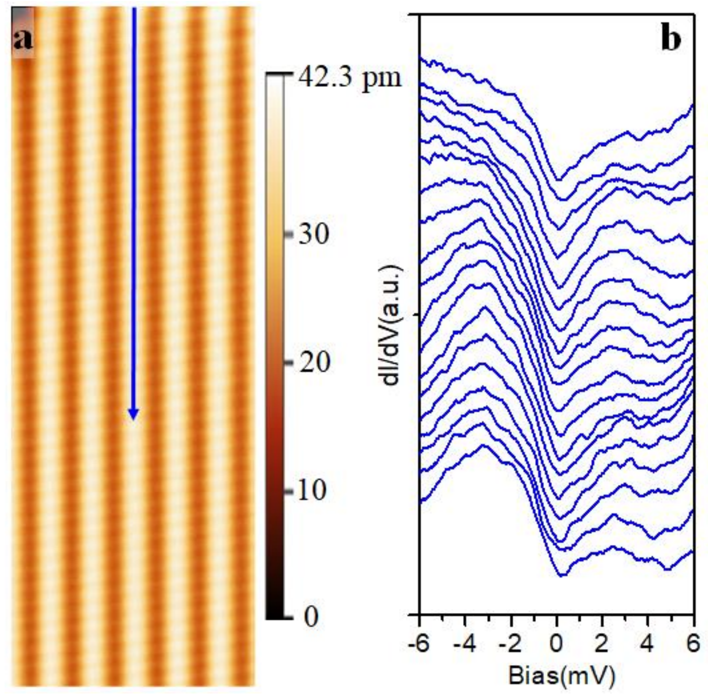

FIG. S3. (a) STM image shows a surface area, image size: 10 nm × 30 nm. (b) A series of spectroscopic survey taken along the blue line in (a) panel. All d*I*/d*V* tunneling spectra are measured at 0.4 K with a bias voltage of -10 mV, a tunneling current of 500 pA. The bias modulation is set at 300 μV.

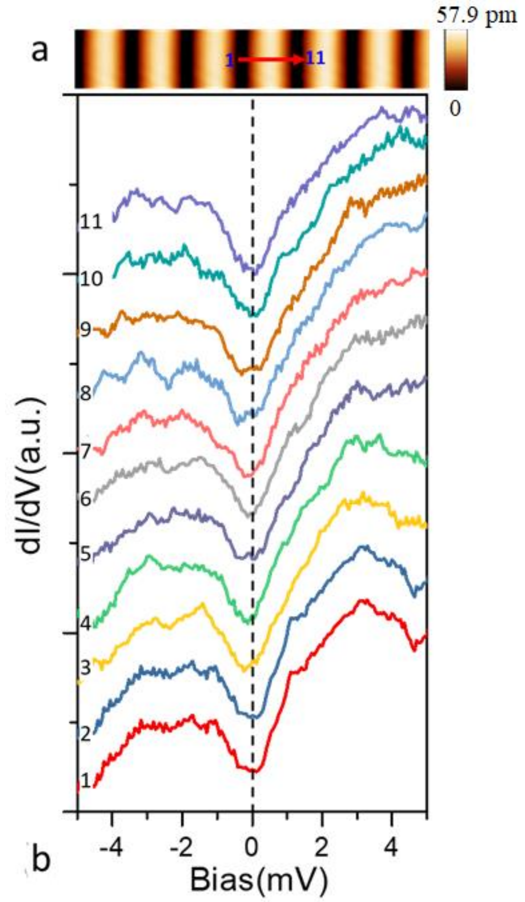

Fig. S4. (a) STM image shows a surface area with periodical 1D chains. (b) A series of spectroscopic survey taken along the red arrow in (a) panel by across a 1D chain.

Supplementary discussions on exclusion of other possible mechanisms of the STS gap formation

1. Exclusion of pseudogap

In such a narrow temperature range of 0.4-1.1 K, extremely small temperature variation can induce significant change of gap shape and eventually gap feature is vanished at T_c, ruling out the possibility of pseudogap which is insensitive to the variation of temperature.

2. Exclusion of charge density wave (CDW) gap

By high-resolution STM measurements, CDW is not observed in atomically resolved images at 0.4 K and 4 K, suggesting that the gap should not be a CDW gap.

3. The STS and transport measurement results together evidence the observation of superconductivity

The superconductivity of gap feature can be supported by the evolution of tunneling spectra with increasing temperature and external magnetic field (Fig. 3(c)-(d) in main text) respectively, exhibiting a typical behavior of superconducting gap. More convincingly, the critical temperature and critical magnetic field obtained from STS investigation match well with the results of transport measurement as shown in Fig. 4 (main text).

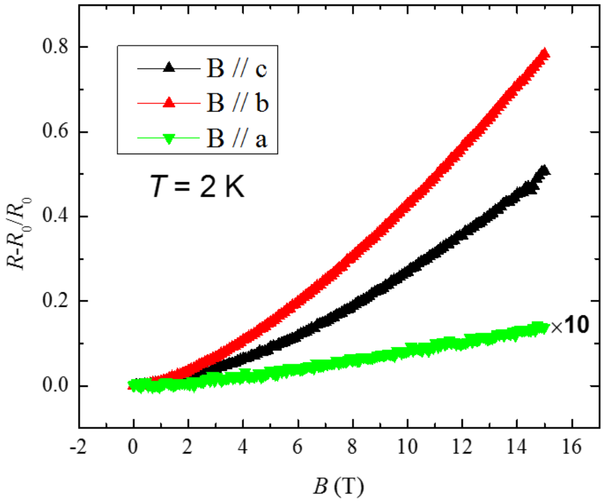

FIG. S5. Magnetoresistance of S1 for three orientations of the magnetic field at 2 K.

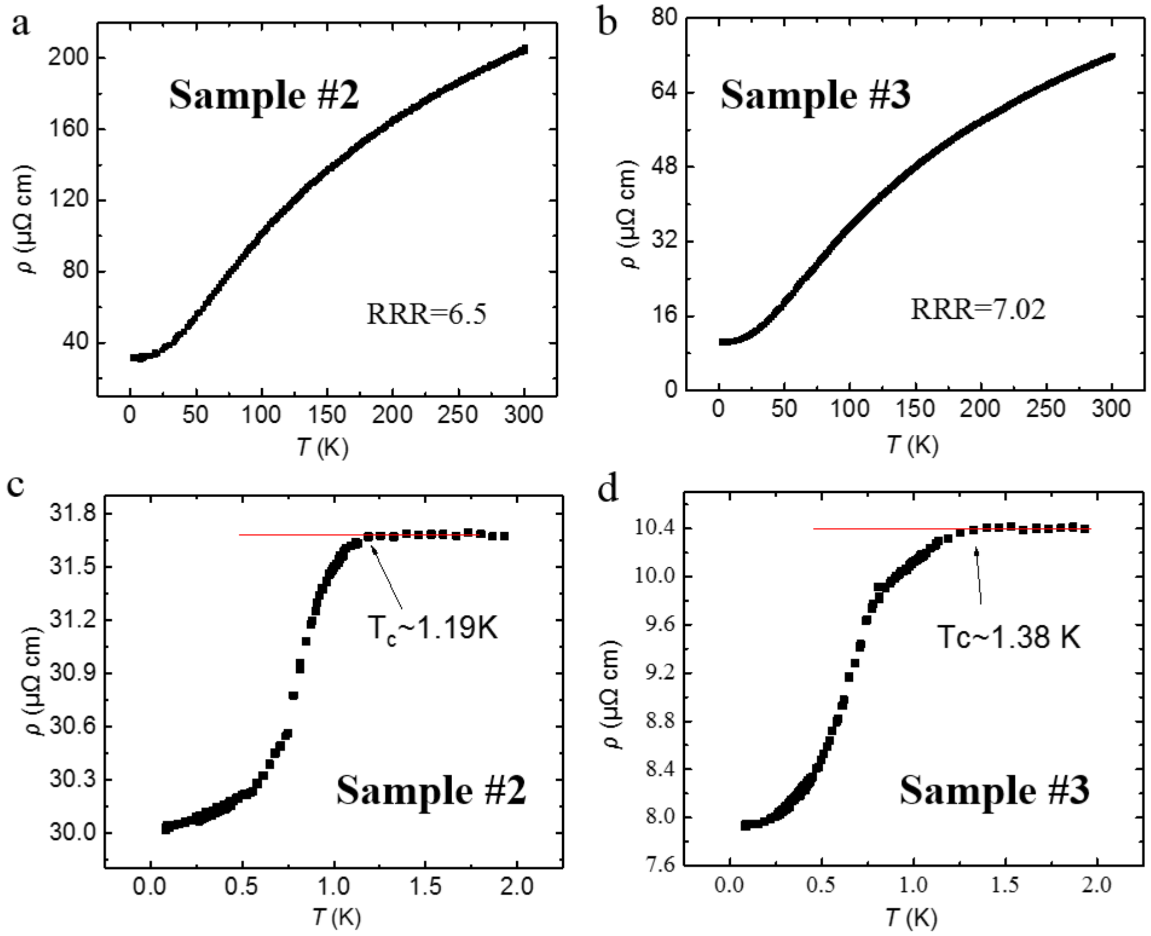

FIG. S6. Superconducting properties of S2, S3. $\rho(T)$ curves of S2(**a**), S3(**b**) from 2 K to 300 K at zero magnetic field. (c), (d) $\rho(T)$ curves below 2 K. The resistivity drops locate at 1.19 K, 1.38 K, respectively.

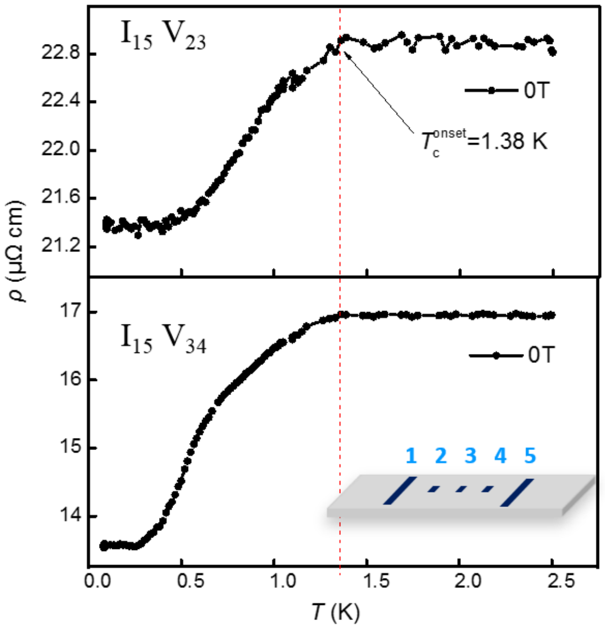

FIG. S7. $\rho(T)$ curves of S3. Five electrodes are made on a long striped $TaIrTe_4$ sample. Standard four-probe method is used to measure the transport property of different regions in one $TaIrTe_4$ sample. The current sides are 1 and 5. The voltage sides are 2, 3 (upper panel) and 3, 4 (lower panel). T_c^{onset} for both regions is similar and about 1.38 K. This excludes the macroscopic superconducting phase separation in $TaIrTe_4$ samples. The inset shows the measurement structure schematic diagram.

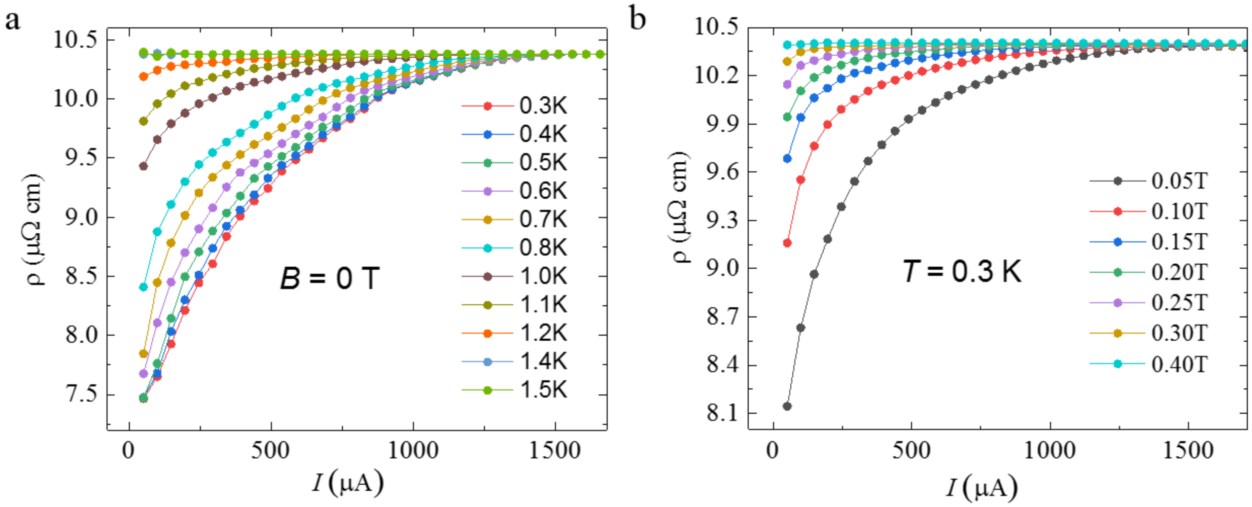

FIG. S8. The sample was obtained by mechanical exfoliation from Sample 3. (a) $R(I)$ curves of 30 μm thick $TaIrTe_4$ measured at temperatures ranging from 0.3 K to 1.5 K at B = 0 T. (b) $R(I)$ curves of 30 μm thick $TaIrTe_4$ measured at magnetic field ranging from 0.05 T to 0.4 T for T = 0.3 K.

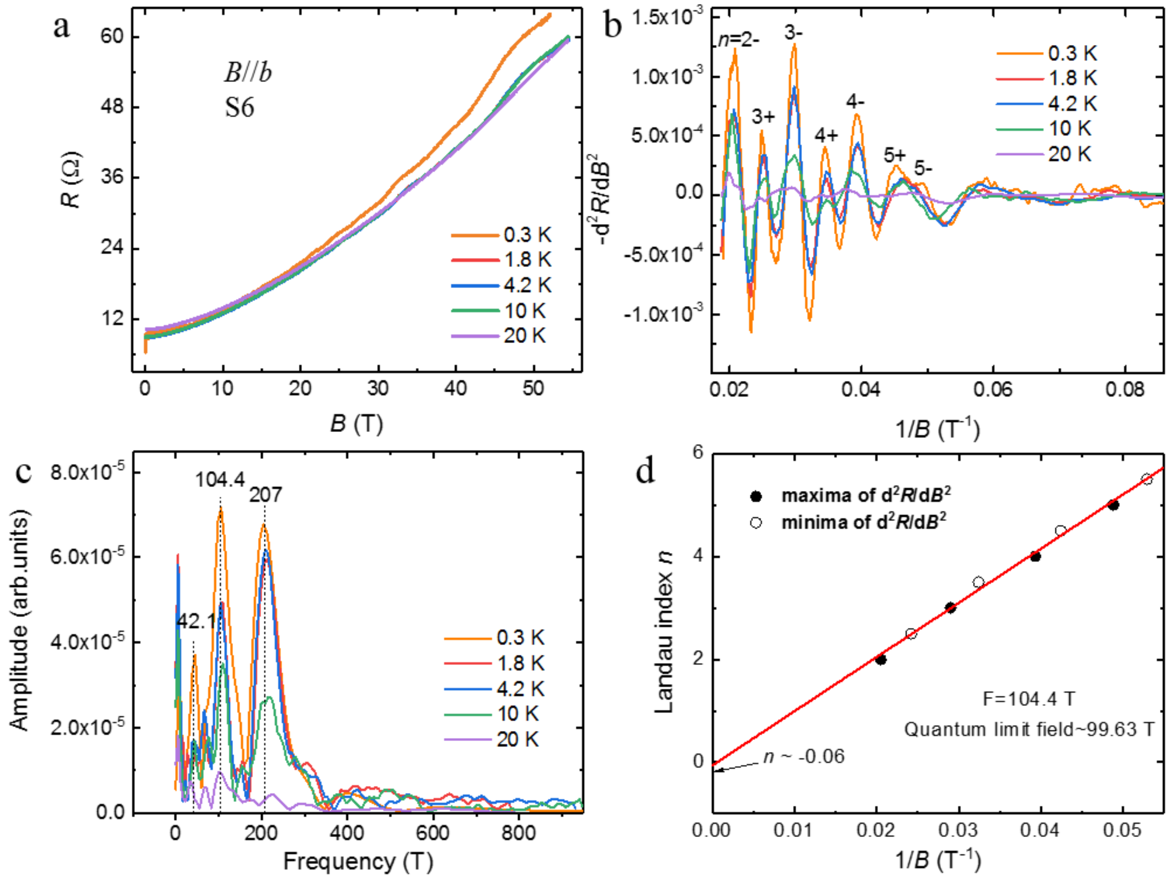

FIG. S9. Quantum oscillations in $TaIrTe_4$ single crystals (S5) with magnetic field perpendicular to the *ac* plane (*B//b*). (a) Magnetic field (up to 54.5 T) dependence of resistivity at different temperatures. (b) Second derivative of the $\rho(B)$ data in (a) vs. $1/B$. (c) FFT analysis with two major frequencies (42.1 T and 104.4 T) for d^2R/dB^2 vs. $1/B$ in (b). (d) Landau level indices (n) as a function of $1/B$.

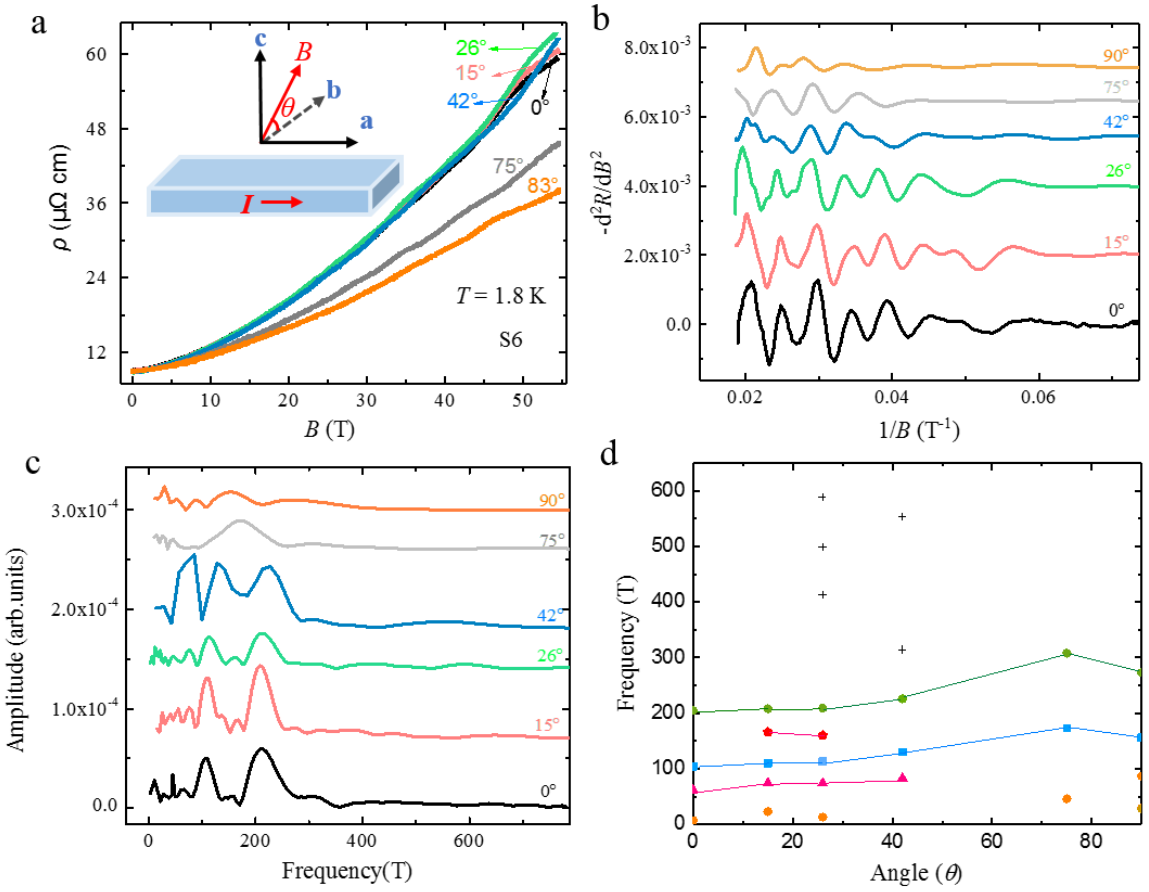

FIG. S10. Angular dependence of SdH oscillations of S5 by rotating the sample from [010] direction to [001] direction at different magnetic field directions. (a) $\rho(B)$ curves at different magnetic field angles from $B_{[010]}$ ($\theta = 0°$, B is parallel to the b axis) to $B_{[001]}$ ($\theta = 90°$, B is parallel to the c axis). (b) Second derivative of the $R(B)$ data in (a) vs. $1/B$. (c) FFT analysis for d^2R/dB^2 vs. $1/B$ in (b). (d) Angular dependence of the SdH frequencies determined in transport data.

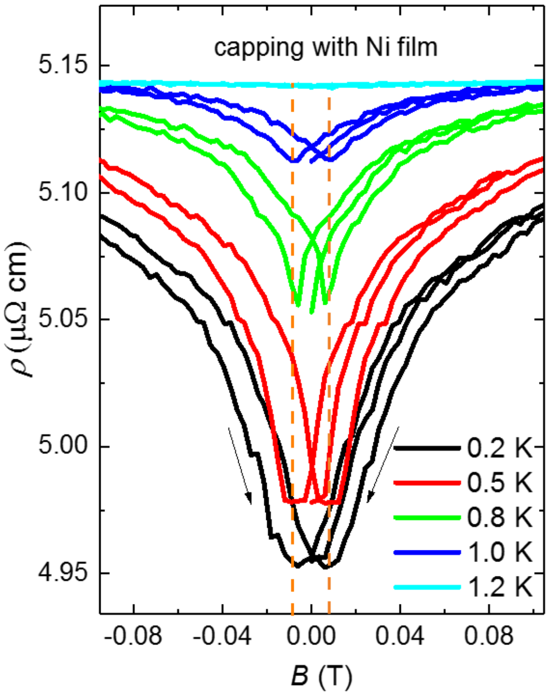

FIG. S11. Magneto-resistance of $TaIrTe_4$ capping with Ni film from 0.2 K to 1.2 K reveals the hysteretic behavior in different sweep directions. The black arrows indicate the magnetic field sweep direction.

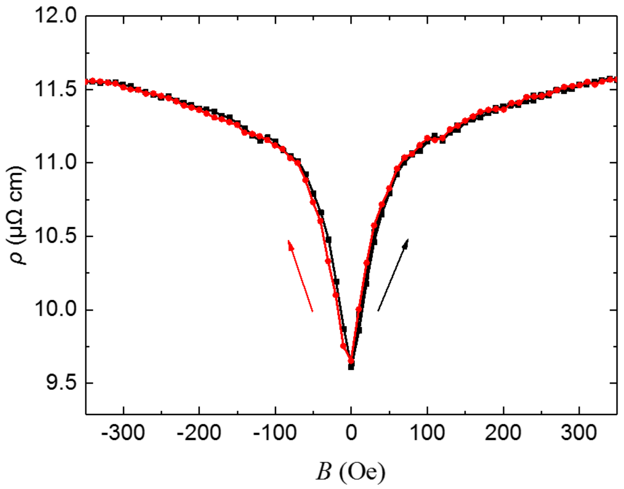

FIG. S12. Magnetoresistance of S6 at 0.5 K. No oscillation and hysteretic behavior was found from different sweep directions at low field region. This excludes the possibility of magnetic phase transitions in $TaIrTe_4$ at low temperature.

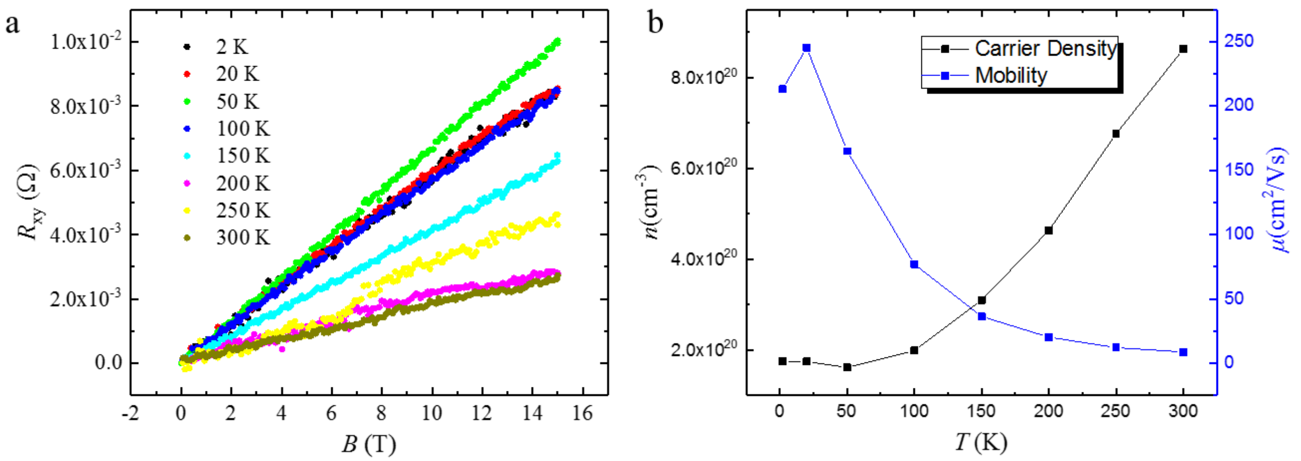

FIG. S13. Hall results of S1. (a) Hall resistance (R_{xy}) varies with magnetic field at different temperatures from 2 K to 300 K. The field is applied perpendicular to the *ab* plane ($B//c$) and the current is along the *a*-axis of the orthorhombic crystal structure ($I//a$). Any additional resistance due to the misalignment of the voltage leads or thermal effect has been removed by averaging the R_{xx} data over positive and negative field directions. (b) Calculated carrier density (n) and mobility (μ) at different temperatures.

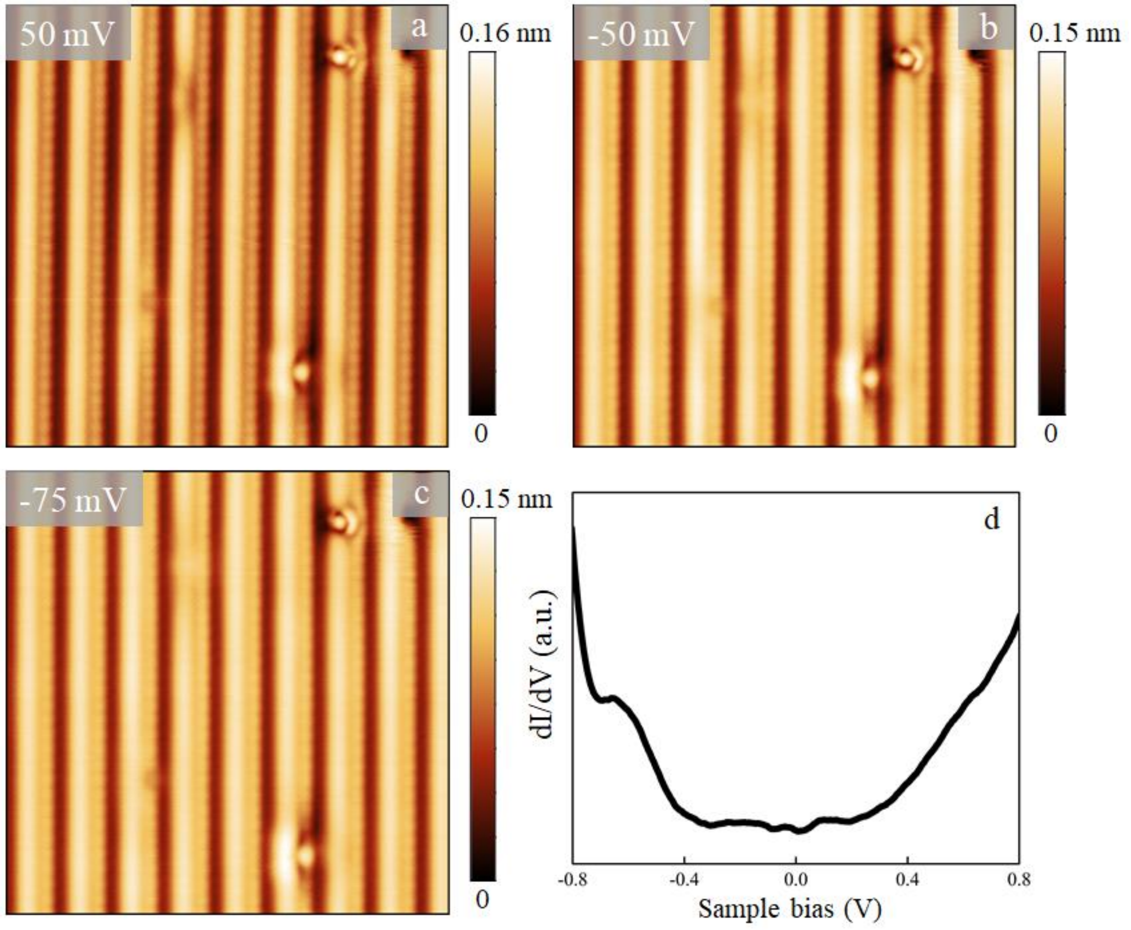

Fig. S14. (a)-(c) The atomically resolved images acquired at different bias. Size: 10×10 nm^2, I_t=300 pA. (d) Large-energy-scale dI/dV spectrum. The bias modulation and tunneling current are set at 8 mV and 200 pA respectively.

The experimental evidences on surface superconductivity in $TaIrTe_4$ are summarized as **Table I**.

**Conclusion**	**Experimental evidence**	**Note**
Superconductivity	Superconducting gap which can be suppressed by increasing magnetic field and temperature (Fig.3)	Evidence of superconductivity
	Superconducting resistance drops which can be suppressed by increasing magnetic field, temperature and current (Fig.4b, Fig.4c, Fig.5a, Fig. S8)	Evidence of superconductivity
Homogeneity of superconductivity	XRD(Fig.1a), STEM(Fig.1b) , STM(Fig.1d,1e,Fig.3a)	Evidence of high quality single crystal
	Multi-electrodes transport results (Fig.S7)	Exclude macroscopic phase separation
	Uniform superconducting gap in whole surface(Fig.S3)	Exclude minority phase region
The superconductivity comes from the surface state.	Nearly thickness-independent critical current. (Fig. 5a)	Fully exclude the possibility of bulk superconductivity
	The angular dependence of the upper critical field (Fig.5b)	A cusp-like peak is qualitatively distinct from the 3D model
	The superconducting gap is detected by surface sensitive STM (Fig.3, Fig.S3 and Fig.S4)	The sample surface is superconducting.
The possibility of nontrivial topological superconductivity	Fermi arc states observed by STS (Fig.2)	Topological non-trivial surface state
	Ferromagnetic Ni film has little effect on the onset T_c of $TaIrTe_4$ (Fig.5c,Fig.S11)	Suggesting the possibility of topological superconducting paring symmetry in $TaIrTe_4$
	$h^*(T/T_c)$ relation is close to that of a polar *p*-wave state (Fig. 4c inset)	Suggesting the possibility of topological superconducting paring symmetry in $TaIrTe_4$
	Superconducting gap becomes smaller and shallower by approaching to the broken end (Fig. 3f)	Suggesting that the pairing order might be unconventional
Quasi 1D feature	STEM (Fig.1b) , STM (Fig.1d, Fig.1e, Fig.3a)	Quasi-1D structure
	Fermi arc states observed by STS (Fig.2)	Quasi-1D behavior on the surface
	The anisotropy of upper critical field(Fig.4f) and magnetoresistance (Fig.S5)	Quasi-1D superconductivity and magnetoresistance characteristic
